# Ski-interacting protein (SKIP) interacts with androgen receptor in the nucleus and modulates androgen-dependent transcription

**DOI:** 10.1186/1471-2091-14-10

**Published:** 2013-04-08

**Authors:** Daniel Abankwa, Susan M Millard, Nick Martel, Catherine S Choong, Miao Yang, Lisa M Butler, Grant Buchanan, Wayne D Tilley, Nobuhide Ueki, Michael J Hayman, Gary M Leong

**Affiliations:** 1 The University of Queensland, Obesity Research Centre, Institute for Molecular Bioscience, St.Lucia, Queensland, 4072, Australia; 2 Department of Paediatric Endocrinology and Diabetes, Mater Children’s Hospital, South Brisbane, Queensland, 4010, Australia; 3 Department of Paediatric Endocrinology and Diabetes, Princess Margaret Children’s Hospital, School of Pediatrics and Child Health, University of Western Australia, Subiaco, Western Australia, 6008, Australia; 4 Dame Roma Mitchell Cancer Research Laboratories and Adelaide Prostate Cancer Research Centre, School of Medicine, University of Adelaide and Hanson Institute, Adelaide, South Australia, 5005, Australia; 5 Department of Molecular Genetics and Microbiology, Stony Brook University, Stony Brook, New York, 11794-5222, USA; 6 Current addresses: Turku Centre for Biotechnology, Åbo Akademi University, Turku, Finland; 7 Head, Cancer Biology Group, Freemasons Foundation Centre for Men’s Health, Basil Hetzel Institute for Translational Health Research, University of Adelaide, Adelaide, South Australia, 5005, Australia

## Abstract

**Background:**

The androgen receptor (AR) is a member of the nuclear receptor (NR) superfamily of ligand-inducible DNA transcription factors, and is the major mediator of male sexual development, prostate growth and the pathogenesis of prostate cancer. Cell and gene specific regulation by the AR is determined by availability of and interaction with sets of key accessory cofactors. Ski-interacting protein (SKIP; SNW1, NCOA62) is a cofactor shown to interact with several NRs and a diverse range of other transcription factors. Interestingly, SKIP as part of the spliceosome is thought to link mRNA splicing with transcription. SKIP has not been previously shown to interact with the AR.

**Results:**

The aim of this study was to investigate whether SKIP interacts with the AR and modulates AR-dependent transcription. Here, we show by co-immunoprecipitation experiments that SKIP is in a complex with the AR. Moreover, SKIP increased 5α-dihydrotestosterone (DHT) induced N-terminal/C-terminal AR interaction from 12-fold to almost 300-fold in a two-hybrid assay, and enhanced AR ligand-independent AF-1 transactivation. SKIP augmented ligand- and AR-dependent transactivation in PC3 prostate cancer cells. Live-cell imaging revealed a fast (half-time=129 s) translocation of AR from the cytoplasm to the nucleus upon DHT-stimulation. Förster resonance energy transfer (FRET) experiments suggest a direct AR-SKIP interaction in the nucleus upon translocation.

**Conclusions:**

Our results suggest that SKIP interacts with AR in the nucleus and enhances AR-dependent transactivation and N/C-interaction supporting a role for SKIP as an AR co-factor.

## Background

SNW/SKIP proteins, which include human Ski-interacting protein (SKIP; SNW1, NCOA62) and its yeast homologue the essential splicing factor Prp45 (Pre-mRNA Processing 45) [1], are phylogenetically highly conserved [2], and important during early development [3,4]. SKIP was identified by its interaction with the proto-oncogenes, v-Ski and c-Ski [5]. In addition to pre-mRNA splicing in the spliceosome [6], SKIP appears to have multiple other functions in transcription [2]. It acts as a transcriptional co-regulator of a number of key cellular signalling molecules, such as of CREB binding protein (CBP)/ p300, the nuclear co-repressor (N-CoR) and silencing mediator for retinoic acid and thyroid hormone receptors (SMRT) [7]. It also interacts with a large range of DNA binding proteins, including Smad2 and 3 proteins of the TGF-β pathway [8], and proteins involved in MyoD and Notch signalling [2] and may be involved in Wnt signalling [9]. A recent study suggests SKIP regulates the cell cycle arrest factor p21 (Cip1) and subsequent effects on p53-dependent DNA cell damage [10]. This appeared to involve recruitment of SKIP to the p21 promoter where SKIP plays a critical role for splicing and p21 gene expression, suggesting a role of SKIP in cancer cell apoptosis.

The fundamental effects of SKIP in transcriptional regulation are supported by its co-regulatory effect on nuclear hormone receptors, including estrogen receptor [11], the Vitamin D receptor (VDR) and Retinoid X Receptor (RXR) [12-15], which it antagonistically regulates in association with SIRT1 [16]. More recently SKIP was shown to associate with P-TEFb, c-myc and Menin to act on the HIV-1 promoter [17,18].

The androgen receptor (AR) is a member of the nuclear hormone receptor superfamily that regulates male sexual development, and is a major player in the pathogenesis and progression of prostate cancer [19-22]. Upon binding to its natural ligands testosterone and 5α-dihydrotestosterone (DHT), the AR becomes phosphorylated and translocates into the nucleus, where it binds as a homodimer to canonical nuclear receptor inverted repeat DNA response elements to activate target gene transcription [23]. A comprehensive ChIP-on-chip analysis of AR-responsive elements (ARE) on chromosomes 21 and 22 however suggests that the majority of the 90 identified binding sites are non-canonical, with isolated half-sites, head-to-head, head-to-tail and direct repeat AREs [24].

The AR contains distinct structural domains also found in other nuclear receptor superfamily members. The amino (N)-terminal transactivation domain, which is also termed ligand-independent activation-function-1 domain (AF-1), is followed by a DNA-binding domain (DBD) that is linked via a hinge region to a carboxy (C)-terminal ligand-binding domain (LBD), which contains the activation function-2 domain (AF-2) [25,26]. Ligand binding induces interaction between the N-terminal and C-terminal domains of AR [27,28]. This interdomain rearrangement slows ligand dissociation and AR degradation [29]. Moreover, mutations in the AR AF-2 domain, which are associated with partial or complete androgen insensitivity syndrome, also abrogate N/C-interaction *in vitro*, suggesting that N/C-interaction is functionally important *in vivo*[27]. The AR intrinsic N-terminal FXXLF (residues 23–27) and WXXLF (residues 433–437) motifs form amphipathic α-helices that stabilise the N/C-interaction, by binding to a hydrophobic pocket at the C-terminus [30]. Importantly, the intrinsic (F/W)XXLF motifs compete with the similar, extrinsic LXXLL-motif from p160-family type I co-regulators for the hydrophobic pocket on the AR C-terminus [31]. Examples for type 1 co-regulators are steroid receptor co-activators (SRC1 and SRC3), transcriptional intermediary factor 2 (TIF2) and amplified in breast cancer-1 (AIB1) [25,32].

While AF-1 mediates hormone-independent constitutive transactivation when artificially isolated, the AF-2 is inactive in the absence of hormone, but required for strong, ligand-dependent activity in androgen-dependent prostate cancer cells [33-36]. This may be due to the fact that even in the absence of ligand the extended AF-1 fragment (including the DBD) assumes a similar reticular or speckled distribution to nuclear foci, as the full-length receptor with its co-regulators, while the isolated AF-2 is distributed homogenously in the nucleoplasm, even if ligand is added [37]. This speckle-pattern distribution is typical for markers of various nuclear compartments, such as speckles, nucleoli and cajal bodies [38,39]. Nuclear speckles are sites of pre-mRNA splicing and SKIP was found among other splicing proteins enriched in nuclear speckles [40].

In this study, we demonstrate that SKIP interacts with the AR. We show that SKIP acts in multiple ways, by augmenting AR AF-1-dependent activity as a classical type I co-activator, while it also enhanced AR N/C-interaction and AR-dependent transcription in prostate cancer cells.

## Results

### Mammalian one-hybrid data show that SKIP augments ligand-independent AR transcription

**Figure 1:**
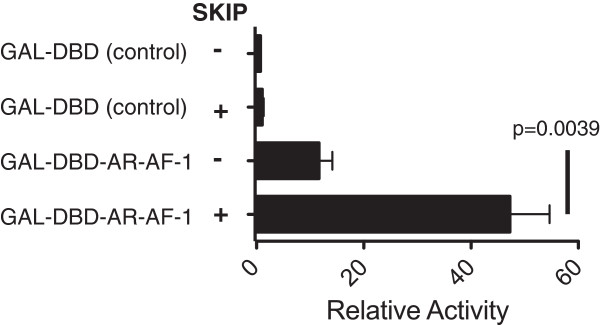
**SKIP augments AR-AF-1 dependent transactivation.** Mammalian one-hybrid analysis in HEK293 cells that were transiently co-transfected with the GAL4_5_E1bTATA-luciferase reporter (250 ng/well), with AR 1–555 N-terminal domain in pM-GAL4DBD expression vector (125 ng) or empty pM-GAL4DBD vector (125 ng), as well as with SKIP-pCGN (100 ng) or empty pCGN (100 ng). To allow for comparison to the N/C-interaction transfection experiments (Figure 2) the empty vector pVP16AD containing the VP16 transactivation domain was also transfected (125 ng). Data are relative to the control sample (no SKIP, no AR-AF1) as means ± SEM calculated from four independent luciferase reporter experiments, as described in Materials and Methods. Statistically significant difference between indicated means was determined using Student’s t-test.

SKIP modulates the transcriptional activity of a variety of nuclear hormone receptors. We therefore tested, whether SKIP can also act as a co-regulator of androgen receptor (AR), which is critically involved in prostate cancer [19,22]. To address, whether SKIP influences AR-mediated transactivation, we used a mammalian one-hybrid assay, which assesses the transcriptional co-activating property of a candidate protein (e.g. SKIP) on a potential interaction partner (e.g. AR) fused to the GAL4 DNA binding domain. The presence of the co-activator increases binding of the fusion protein to GAL4-binding sites on the reporter plasmid, which then drives luciferase transcription [41]. We expressed in HEK293 cells a fragment comprising the N-terminal ligand-independent activation-function-1 (AF-1) domain of AR (residues 1–555) fused to the GAL4 DNA binding domain (AR1-555) with and without SKIP. In the absence of the AR1-555 fusion protein, overexpression of SKIP did not induce reporter gene expression compared to control cells co-transfected with empty vectors (Figure 1). Consistent with the ligand-independent transcriptional activity of the AR AF-1 domain [42], transfection of the AR1-555 fusion protein induced a 10-fold increase in basal, ligand-independent luciferase activity compared to control levels. This AR-AF-1 transcriptional activity was increased ~5-fold by overexpression of SKIP (Figure 1). These data suggest a co-stimulatory activity of SKIP by a functional interaction with the N-terminal ligand-independent activation-function-1 (AF-1) domain of AR.

### SKIP facilitates interaction of AR N- and C-termini

AR transcriptional activity is modulated by ligand-mediated interdomain interaction of its N- and C-termini [27,30]. To address, whether SKIP facilitates AR N- and C-terminal, ligand-dependent interaction, the mammalian two-hybrid system was used [43,44]. In the mammalian two-hybrid system, potentially interacting protein fragments are fused to either the GAL4 DNA binding domain (GAL4-DBD) or the V16 transactivation domain. Interaction of the fused proteins brings the GAL4-DBD and V16 domain into a complex, which increases transcription of luciferase from a GAL4 responsive promoter. Residues 1–538 of AR were fused to the VP16 activation domain and C-terminal AR-LBD residues 642–917 to the GAL4 DNA binding domain (AR642-917) to detect N/C-terminal interaction. Modulation of this interaction by SKIP would alter reporter gene expression in HEK293.

**Figure 2:**
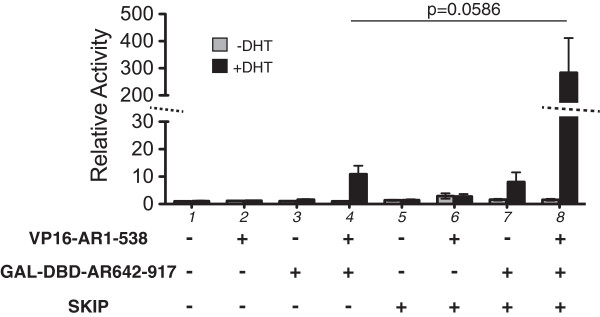
**SKIP increases N- and C-terminal interaction of AR.** Mammalian two-hybrid analysis in HEK293 cells that were transiently co-transfected with the GAL4_5_E1bTATA-luciferase reporter (250 ng/well), and as indicated, GAL4-DBD-fusion constructs and VP16-fusion constructs of the C- or N-terminus of AR (GAL4DBD-AR 642–917 (125 ng each) and pVP16-AR1-538 (125 ng), respectively) with SKIP-pCGN (100 ng) or empty pCGN (100 ng). Reporter activity was determined in cells with (black bars) and without (grey bars) 1 nM DHT stimulation overnight for 16 hours. Response of cells that did not co-express SKIP (columns 1–4), was markedly lower than that of cells expressing SKIP (columns 5–8). Note the discontinuous y-axis for the relative activity. Data are relative to the control sample (empty vectors without DHT stimulation) as means ± SEM calculated from five independent luciferase reporter experiments, as described in Materials and Methods. Statistically significant difference between indicated means was determined using Student’s t-test.

In the absence of SKIP, the AR N- and C-terminal, ligand-dependent interaction resulted in a significant increase in relative luciferase activity by 12-fold (Figure 2 column 4). SKIP also enhanced ligand-dependent activation of the AR-LBD 642–917 construct by 9-fold (Figure 2 column 7). Overexpression of SKIP markedly increased ligand-dependent transcriptional activity mediated by the AR N- and C-terminal constructs to ~300-fold over control levels (Figure 2 column 8 compared with column 4). Thus SKIP facilitates the ligand-induced, interdomain interaction of the N- and C-termini of AR or processes downstream of this rearrangement, augmenting AR transcriptional activity by an order of magnitude.

### SKIP increases AR-dependent transcription in prostate cancer cell lines

**Figure 3:**
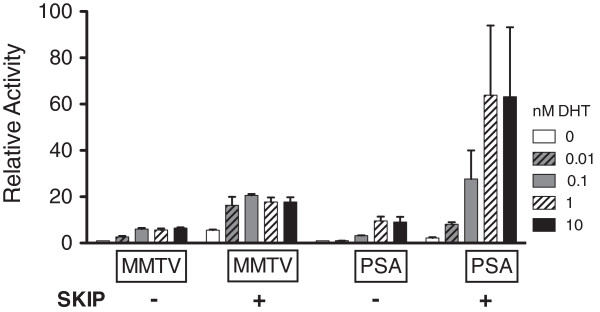
**SKIP augments AR-dependent transcription in prostate cancer cells.** Luciferase reporter transactivation assays were performed in PC3 prostate cancer cells using transiently transfected MMTV- and PSA-luciferase reporters (250 ng/well) with SKIP-pSG5 expression plasmid (200 ng) or empty vector pSG5 (200 ng) as indicated. As PC3 cells have no endogenous AR expression, pCMV-AR WT expression plasmid (25 ng/well) was also transfected in these cells. Data are relative to the no SKIP and no ligand control, which was set as one and expressed as mean ± SEM calculated from 3 independent experiments.

To provide further evidence that SKIP is a co-activator of AR function, the effects of SKIP overexpression on AR–dependent transcription were assessed using the androgen-responsive mouse mammary tumour virus (MMTV) MMTV-luc and the prostate-specific antigen (PSA) PSA-luc luciferase reporter plasmids in a prostate cancer cell line. We used the AR-negative PC3 cell line, where we overexpressed WT AR. In these cells SKIP overexpression augmented basal ligand-independent MMTV reporter activity ~6-fold. Additional stimulation with the AR ligand DHT (0.01 to 10 nM) increased activity ~18-fold over control levels (no SKIP, empty pSG5 vector only, no DHT), without any apparent dose-dependence (Figure 3). On the other hand, SKIP alone induced only a 2-fold increase in basal PSA-reporter activity. This was however markedly and dose-dependently increased by DHT (8-60- fold over control levels), as compared to PC3-cells without co-transfected SKIP (up to 10-fold over control levels).

These results suggest that SKIP augments DHT-induced MMTV- and PSA-reporter activity several-fold, with a response pattern that depends on the reporter gene construct.

### SKIP co-immunoprecipitates with androgen receptor

**Figure 4:**
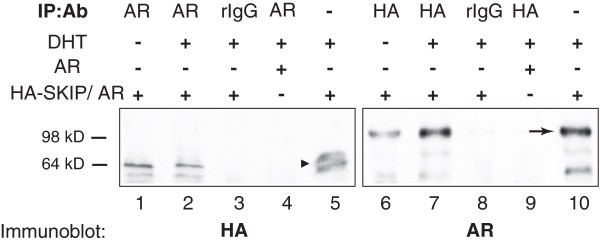
**SKIP and androgen receptor co-immunoprecipitate.** Co-immunoprecipitation experiments in COS-7 cells co-transfected with full-length AR WT plasmid and HA-SKIP-plasmid as indicated and described in Materials and Methods. AR-bound and HA-positive (left) or SKIP-bound and AR-positive (N20-antibody) proteins (right) are shown. rIgG stands for rabbit IgG and represents a negative control. Experiments were carried out in presence or absence of 1 nM DHT, as indicated. Lanes 5 and 10 show blotted lysates as input controls for the immunoprecipitation. Approximate molecular weights are as indicated on the left. Arrow shows AR-fragment positive controls, while arrow-head points to HA-SKIP positive controls. Blots are representative of three independent repeats.

SKIP has been shown to interact with a number of nuclear hormone receptors via its helical repeat domain [2]. We therefore investigated, whether SKIP is also in a complex with AR in a co-immunoprecipitation experiment (Figure 4).

We co-expressed AR with SKIP in COS-7 cells and precipitated bound proteins from the cell lysates using anti-AR or anti-HA antibodies (Figure 4). HA tagged SKIP proteins (~62 kD) were specifically detected in AR-immunoprecipitates in similar amounts, irrespective of whether cells were DHT treated (Figure 4 lanes 1 and 2). Consistent with AR co-immunoprecipitating HA-SKIP, the inverse experiment showed that AR protein (~110 kD) was specifically detected in HA-SKIP immunoprecipitates (Figure 4 lane 6 and 7).

In conclusion, these data suggest that AR and SKIP interact in a specific complex.

### FRET experiments reveal interaction of AR and SKIP in the nucleus

**Figure 5:**
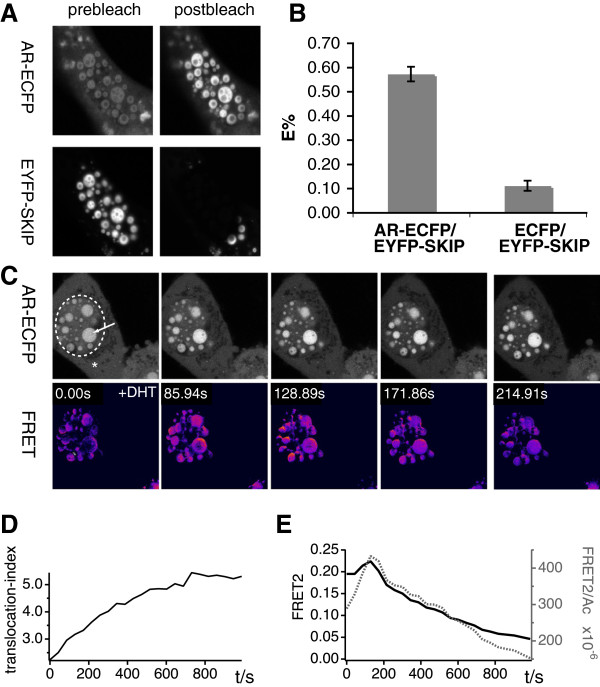
**AR interacts with SKIP after nuclear translocation on SKIP-positive nuclear speckles.** (**A**,**B**) Donor dequenching and (**C**-**E**) sensitised acceptor emission FRET imaging data of AR-ECFP and EYFP-SKIP co-expressed in BHK cells. (**A**) BHK cells were transiently transfected with AR-ECFP and EYFP-SKIP. Before being fixed with 4% PFA, cells were stimulated for 2.5 min with 10 nM DHT at room temperature. The acceptor was then bleached and the increase of the donor-fluorescence was monitored in the donor channel, using confocal imaging. (**B**) The average FRET efficiency of AR-ECFP/EYFP-SKIP (8 images, 24 speckles), E%=57±3% (mean±SEM) was significantly higher (P<0.001, 2-tailed Student’s t-test), than that of the control FRET-pair, ECFP/EYFP-SKIP, E%=11±2% (mean±SEM) (3 images, 9 speckles) on EYFP-SKIP positive nuclear speckles. This is in line with a strong interaction between AR and SKIP on these structures. (**C**) Representative time series of serum starved BHK cells, transiently transfected with AR-ECFP and EYFP-SKIP that were stimulated with 10 nM DHT (+DHT). Using confocal imaging, nuclear translocation of AR-ECFP and FRET-changes (FRET2) were measured over time. Time points are shown in FRET2 images. The approximate boundary of the nucleus is indicated in the first AR-ECFP image. (**D**) The nuclear translocation index of data in **C** was calculated using the largest speckle (arrow in first image) and a region in the cytoplasm (asterisk) (apparent translocation half-time, τ_0.5_=213±9 s). (**E**) The change of the FRET2 signal (FRET2) and the FRET2 signal normalized with the unprocessed acceptor signal (FRET2/Ac) were averaged over the whole nucleus for each time point. Both FRET signals reached their maximum at ~ 129 s after DHT addition, corresponding to ~ 32% translocation. Subsequently the FRET signal decreased, as the fluorescence of EYFP-SKIP disappeared.

Finally, we wanted to monitor AR and SKIP interaction in intact living cells and provide evidence for their direct interaction, using Förster/fluorescence resonance energy transfer (FRET) imaging. We transiently co-expressed AR, which was C-terminally tagged with ECFP (AR-ECFP) as fluorescent donor, and SKIP, N-terminally tagged with EYFP (EYFP-SKIP) as an acceptor in BHK cells (Figure 5). The EYFP-SKIP construct localized to small nuclear structures of various sizes (Figure 5A; Additional file 1). For our FRET experiments, we selected for those cells that contained larger SKIP-positive nuclear structures. Note, that even under serum starvation, cells exhibited various degrees of nuclear co-localization of AR-ECFP and EYFP-SKIP (data not shown). Using donor-dequenching FRET experiments on maximally DHT stimulated BHK cells, we measured a high FRET efficiency of 57±3%, as compared to 11±2% background (Figure 5B).

In order to gain further insight into the temporal characteristics of this interaction, we followed the DHT induced nuclear translocation of AR-ECFP to EYFP-SKIP positive structures in live BHK cells. We used sensitised acceptor emission FRET imaging with fully crosstalk corrected FRET images [45]. When stimulated with DHT (Figure 5C), AR-ECFP accumulated on EYFP-SKIP positive structures of the nucleus within 3 minutes (Figure 5D), with concomitant increase in the FRET signal (Figure 5E; Additional file 2). On individual SKIP-positive nuclear structures, the FRET signal first increased at the periphery and then progressed towards the centre. Thereafter, the FRET signal decreased, due to a drop in the EYFP-SKIP signal, which was not due to photobleaching caused by progressive scanning of the sample (data not shown).

In conclusion, FRET-experiments provide additional evidence that a direct and transient interaction of AR and SKIP occurs within the nucleus, which increases within minutes after DHT-stimulated nuclear translocation of AR.

## Discussion

In this study we provide evidence that SKIP acts as a co-regulator of AR transcription. Using mammalian one- and two-hybrid-experiments, we showed that SKIP augments AR AF-1 ligand-independent transcriptional activity and AR ligand-dependent AF-1 and AF-2 (AR N/ C-terminal) transcriptional interaction. Moreover, SKIP augmented AR-dependent transcription with two ARE-containing reporters in prostate cancer PC3 cells. Immunoprecipitation and FRET-experiments showed that AR interacts with SKIP and that the AR translocates to the nucleus within minutes after ligand stimulation to interact with SKIP.

FRET detects proximities of 3–7 nm for ECFP/EYFP-FRET pairs [46], which is a distance that can in most cases be regarded to report on direct interactions, considering that the size of the fluorescent proteins is already 2 nm × 4 nm [47]. The observed high FRET efficiency between AR-ECFP/ EYFP-SKIP on the speckle patterned SKIP-positive structures is therefore in agreement with a direct interaction of these proteins.

Our FRET data are supported by our one- and two-hybrid data, which suggest that SKIP can activate both the N- and C-terminus of AR. Therefore, FRET-fluorophores on the C-terminus of AR and N-terminus of SKIP may come into very close proximity, despite the relatively large size of both proteins. The strong increase of FRET upon DHT-stimulated nuclear translocation of AR-ECFP to EYFP-SKIP positive structures that resemble nuclear speckles, can be explained by the fact that an excess of the acceptor, EYFP-SKIP, leads to high FRET-levels in a molecular complex, where the donor-acceptor-ratio is 1: >1 [48].

Nuclear speckles form a functional compartment within the nucleus that include splicing factors. Active gene transcription coupled with pre-mRNA splicing is thought to occur at the periphery of the splicing factor compartment [49-51]. It was therefore intriguing to see that the FRET between SKIP and AR increased from the outside to the inside of the speckle-like SKIP-positive structures. However, as fluorescently tagged proteins had to be overexpressed, care must be taken when relating the magnitude of the FRET with the affinity of the two proteins under physiological conditions. On the other hand, overexpression does not seem to induce random interaction in our experiments, as the interaction only significantly increased a) after DHT-stimulation and b) involving a translocation from the cytoplasm to the nucleus. We cannot say, whether AR is in addition targeted to other nuclear compartments.

It is of interest that the AR AF-1 domain has been shown to interact within nuclear speckles with another splicing factor ANT-1 (homologous to yeast splicing factor Prp6p) [52]. This distribution resembles the AR-SKIP co-localization that we have observed (Figure 5). Thus AR interaction with SKIP and SKIP enhancement of AR AF-1 transcriptional activity is similar to AR/ ANT-1-dependent augmentation of transcription and splicing. Therefore, further studies will be required to determine if SKIP, like ANT-1 may recruit AR into a transcription-splicing coupled machinery.

Interestingly, the SKIP-EYFP signal disappeared completely ~16 minutes after DHT mediated translocation of AR to the nucleus, suggesting AR-mediated degradation or masking of SKIP. Loss of the SKIP-EYFP signal started soon after the maximum FRET was reached, raising the possibility that a regulated degradation is triggered at high AR concentrations. The AR has been previously linked to proteasome-mediated degradation, by its interaction with ubiquitin protease USP10 [53], the ubiquitin ligase ARNIP [54] and sensitivity of AR transactivation to proteasome inhibitors [55].

AR transcriptional activity is dependent on its N-terminal and C-terminal domains, which if artificially isolated can act in a ligand-independent (AF-1) and ligand-dependent (AF-2) manner, respectively [23,34]. Immunoprecipitation and FRET-data allow us to explain the transactivation data by the direct interaction of SKIP with AR, however details of the exact mechanism are still unclear. We showed that the AR N-terminal domain was sufficient to engage SKIP to increase transactivation. It is possible that in a transcriptionally active complex, such as that induced by the N-terminal AR fragment, SKIP is engaged in the recruitment of general co-regulators such as N-CoR and p300 [7]. In line with the latter interpretation, it was previously found that AR ligand-dependently interacts with the nuclear receptor co-repressor, N-CoR [56], which also interacts with SKIP [7].

Moreover, our two-hybrid data indicate that SKIP bi-functionally interacts with the N- and C-termini of AR, or at least facilitates this N/C- interaction. Interestingly, Saitoh et al. [37] observed something similar for the effect of the co-regulator CBP, which is also required for nuclear foci or speckle formation. In addition, SRC1 also modulates N/C-terminal interactions [57], however, this and its co-regulator activities are handled by distinct parts of the protein with an apparently higher significance for its LXXLL-motif independent interaction [58]. Moreover, co-regulators were shown to modulate the N/C-interaction, in a cell type dependent manner. Correspondingly, their effect on the N/C-interaction did not always correlate with their effects on AR-mediated transactivation or cell growth, which may highlight the importance of specific microenvironments that influence this interaction [57]. This may be explained by the assumption that the AF-1 may be the major transactivator under normal physiological conditions, while type I co-regulators come into play, if their concentration is high, such as observed in recurrent prostate cancer [28].

## Conclusions

In conclusion, our data suggest SKIP as a novel AR co-activator that enhances its AF-1 function and interdomain-interaction, as well as AR-dependent transcription. The diverse network of interactions shown for SKIP, in particular with nuclear hormone receptors, explains its impact on several signalling pathways involved in growth and development, including possibly in AR-dependent prostate cancer cell growth.

## Methods

### Reporter gene assays

HEK293 cells (origin: *H. sapiens*: embryonal kidney cells); PC3 (origin: *H. sapiens:* prostate cancer cells) were maintained in medium with 10% foetal calf serum and plated 24–36 hours before transfection into 24 well plates at a density of 2×10^4^ cells (HEK293) or 2.5×10^4^ cells (PC3) per well so that at time of transfection cells were about 75% confluent. Cells were transfected overnight with Fugene 6 (Roche) following the manufacturer’s instructions, using 1.5 μl Fugene per 0.6 μg of total plasmid DNA per well. The ligand DHT in DMEM/F12 for HEK293 cells of RPMI for PC3 cells was added to medium supplemented with 2% charcoal stripped FCS for 24 hours. Medium was removed and cells were washed once with ice cold PBS and then lysed with 2× Promega lysis buffer. Luciferase assays were performed in triplicate with the Firefly luciferase assay kit (Promega) and measured on a luminometer (Berthold LB953 Autolumat), as described previously [7]. The PC3 cell line expresses no endogenous AR [59].

### Co-immunoprecipitation and western blotting

Co-immunoprecipitations were performed as follows. COS-7 cells (origin: *Cercopithecus aethiops,* African green monkey, derived from CV-1 cells by SV40 mediated immortalization [60]) cultured under standard conditions in 10 cm culture dish (NUNC) were co-transfected using Lipofectamine™ 2000 (Invitrogen) with pCMV-AR and/or SKIP-pCGN plasmid DNA (10 μg each) and cultured in the presence or absence of 1 nM DHT. Cells were harvested 48 h after transfection and were lysed in 0.5 ml of cold RIPA buffer (50 mM Tris, pH 8.0, 150 mM NaCl, 1 mM EDTA, 0.1% SDS, 0.5% deoxcholic acid, 1% Triton X-100) containing 1 mM PMSF and proteinase inhibitors (Roche). Lysates were sonicated for 30 seconds on ice and centrifuged to remove cell debris then pre-clarified by incubation with 40 μl of 50% protein G-Sepharose beads (Amersham) and 1 μg of rabbit IgG (Dako) at 4°C for 1 hour with slow rotation to reduce non-specific binding. After pelleting the beads, the lysates were incubated with rabbit anti-AR antibody, N20 (Santa Cruz Biotechnology), and rabbit anti-HA antibody (Santa Cruz Biotechnology), respectively, at 4°C overnight, and followed by incubating with 20 μl of 50% protein G-Sepharose beads at 4°C for 1 hour to recover AR or HA-SKIP containing protein complexes. The immunoprecipitates were washed 5 times with cold RIPA buffer and resuspended in 20 μl of sample buffer before subjecting to western blotting with antibodies to AR or HA-SKIP. Briefly, parts of the immunoprecipitated sample was resolved on an 8% SDS-PAGE, blotted and probed for HA-tagged SKIP using anti-HA antibody. The other parts were separated on gels, blotted and probed with anti-AR antibody, N20 to detect an AR N-terminal fragment. The probed blots were incubated with anti-rabbit IgG HRP (Amersham) and imaged using ECL (Amersham).

### Plasmid constructs

SKIP-pSG5, SKIP-pM, SKIP-pCGN were previously described [7,8]. All mammalian one and two-hybrid plasmids (pM-GAL4DBD and pVP16AD; Clontech State, USA) and the GAL4_5_E1bTATA-luciferase reporter [61] were previously described [7]. The MMTV-luciferase reporter MMTV-luc was provided by Dr. R.M. Evans, Salk Institute, La Jolla, CA and the PSA-luciferase reporter plasmid (pGL3-PSA540-enhancer, PSA-luc) provided by Bristol-Myers Squibb (Princeton, NJ) as previously described [62]. The vector pM-GAL4DBD-AR-AF1 expressed the activation function 1 fragment of AR (residues 1–555). The vectors pM-GAL4DBD-AR642-917 and pVP16-AR1-538 were as described [41]. pCMV-AR WT-pCMV3.1 plasmid for co-immunoprecipitation experiment was previously described [63,64]. All AR constructs are derived from human AR. EYFP-SKIP human Skip cDNA was amplified by PCR using primer pairs (5^′^-TTT GAA TTC ATG GCG CTC ACC AGC TTT TTA CCT GC-3^′^ and 5^′^-TTT GTC GAC TAT TCC TTC CTC CTC TTC TTG CC-3^′^), phosphorylated by T4 polynucleotide kinase, and cloned into the SmaI site of pEYFP-C1 (Clontech). The AR-pECFP-N1 plasmid was as previously described [37].

### Donor dequenching and sensitised acceptor FRET imaging experiments

FRET experiments were performed in BHK-21 cells (origin: *Mesocricetus auratus,* golden hamster) that were cultured and transfected as previously described. BHK cells were chosen as they were easy to transfect and showed good expression of the constructs. Donor dequenching experiments were carried out, using a LSM 510 Meta confocal microscope (Zeiss). Fluorescent 12 bit images were recorded in the donor- (ex 405 nm, em 530–600 nm) and acceptor- (ex 514 nm, em 530–600 nm) channels before and after bleaching of EYFP (at 514 nm with 100% laser transmission at 50% output in a ROI circumscribing nuclear speckles using 200 iterations). Average bleaching of the EYFP-signal was 98.3±0.5% (sem). The average apparent donor dequenching FRET-efficiency was calculated on individual EYFP-SKIP positive speckles, as: E=1-Dbefore/Dafter, where Dbefore/after are the background corrected average donor channel signals before and after bleaching, respectively. On each recorded image 3 different SKIP-positive speckles were analysed. Sensitized acceptor emission FRET imaging was carried out essentially as described [45]. However, due to the inherently large difference in donor and acceptor expression, only the FRET index image, FRET2 [65], was calculated for each time point. The colour-lookup-table was assigned to the FRET2-images using Image J. The video showing the donor, FRET and acceptor-images was generated using Image J and Quick Time Pro. The translocation index, TL, was calculated on the indicated regions using the intensity of the AR-ECFP on the speckle, S, and the AR-ECFP intensity of a reference region in the cytoplasm, C: TL=S/C. For graphical representation and curve fitting, this value was then normalized using the TL at time 0 and at the last time point 988 s: TL_norm_=(TL-TL_0_)/(TL_988_-TL_0_). To determine the translocation half-time, τ_0.5_, where 50% translocation occurred, a monoexponential function was fitted: TL_norm_=1-exp(−t/τ), with τ_0.5_= − τ ln0.5.

## Authors’ contributions

DA, SMM, NM, CSC, MY, LMB, GML conceived and designed the experiments; DA, NM, CSC, MY, LMB, GB, GML analyzed the data; DA, SMM, NM, CSC, MY, LB, WDT, NU, MJH, GML contributed reagents/materials/analysis tools; DA, GB, GML wrote the manuscript. All authors read and approved the final manuscript.

## Supplementary Material

Additional file 1 Confocal imaging data of EYFP-SKIP showing its diverse speckled distribution patterns in BHK cells. Click here for file

Additional file 2 **The video shows the donor- (left, green), calculated FRET- (middle, colour coded), and the acceptor-images (right, yellow).** DHT was added in frame 1 (time given in middle panel) at 10 nM. The donor images show a DHT induced increase in AR-ECFP translocation from the cytoplasm to the EYFP-SKIP positive nuclear speckles. This leads to a transient increase in FRET on the speckles, suggesting an increased number of AR/SKIP interactions. The FRET signal subsequently decreased, as the signal of EYFP-SKIP diminished. Click here for file
