# Supplementary figures and images for: Ski-interacting protein (SKIP) interacts with androgen receptor in the nucleus and modulates androgen-dependent transcription
Source: BMC Biochem. 2013 Apr 8;14:10. doi: 10.1186/1471-2091-14-10 (PMC3668167; doi:10.1186/1471-2091-14-10)

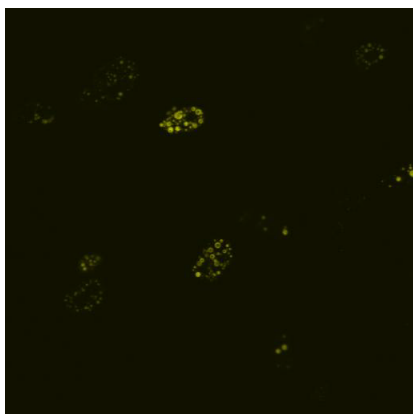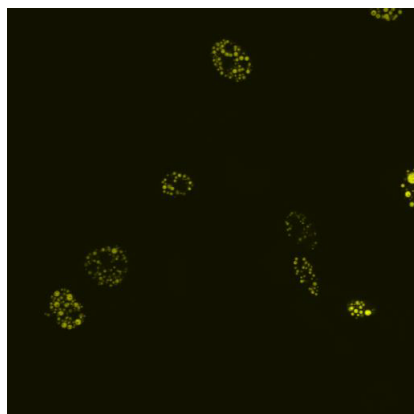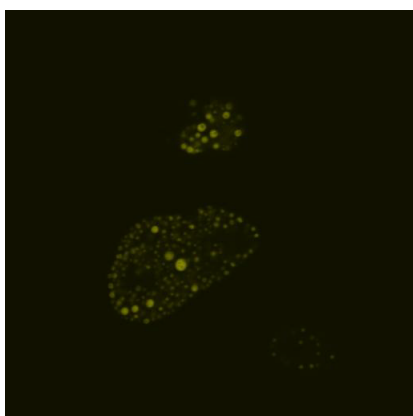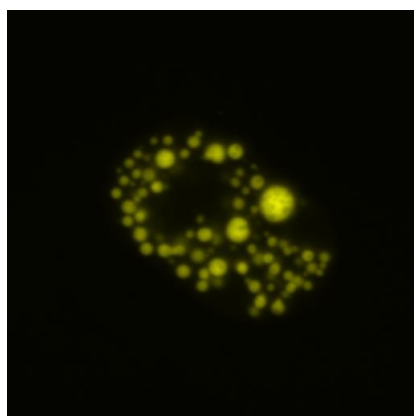

S Figure 1

Supplement: Additional file 1 — Confocal imaging data of EYFP-SKIP showing its diverse speckled distribution patterns in BHK cells. [file 1471-2091-14-10-S1.pdf]
